# Propofol-related urine discoloration in a patient with fatal atypical intracerebral hemorrhage treated with hypothermia

**DOI:** 10.1186/2193-1801-3-551

**Published:** 2014-09-23

**Authors:** Martin Regensburger, Hagen B Huttner, Arnd Doerfler, Stefan Schwab, Dimitre Staykov

**Affiliations:** Department of Neurology, University of Erlangen-Nuremberg, Schwabachanlage 6, 91054 Erlangen, Germany; Division of Molecular Neurology, University of Erlangen-Nuremberg, Schwabachanlage 6, 91054 Erlangen, Germany; Department of Neuroradiology, University of Erlangen-Nuremberg, Schwabachanlage 6, 91054 Erlangen, Germany

**Keywords:** Hypothermia, Intracerebral hemorrhage, Urine discoloration, Enterohepatic circulation, Liver enzyme function

## Abstract

**Introduction:**

Mild therapeutic hypothermia is an increasingly recognised treatment option to reduce perihemorrhagic edema in severe intracerebral hemorrhage.

**Case description:**

We report the case of a 77-year old woman with atypical intracerebral hemorrhage that was treated with mild hypothermia in addition to osmotic therapy. The patient’s urine subsequently showed a green discoloration. Urine discoloration was completely reversible upon discontinuation of propofol.

**Discussion and evaluation:**

Propofol-related urine discoloration may have been provoked by hypothermia. Due to the benign nature of this side effect, propofol should be stopped and gastrointestinal function should be supported.

**Conclusion:**

More studies are needed to show a causal role of hypothermia and related decreased enzymatic function.

## Case

**Figure 1:**
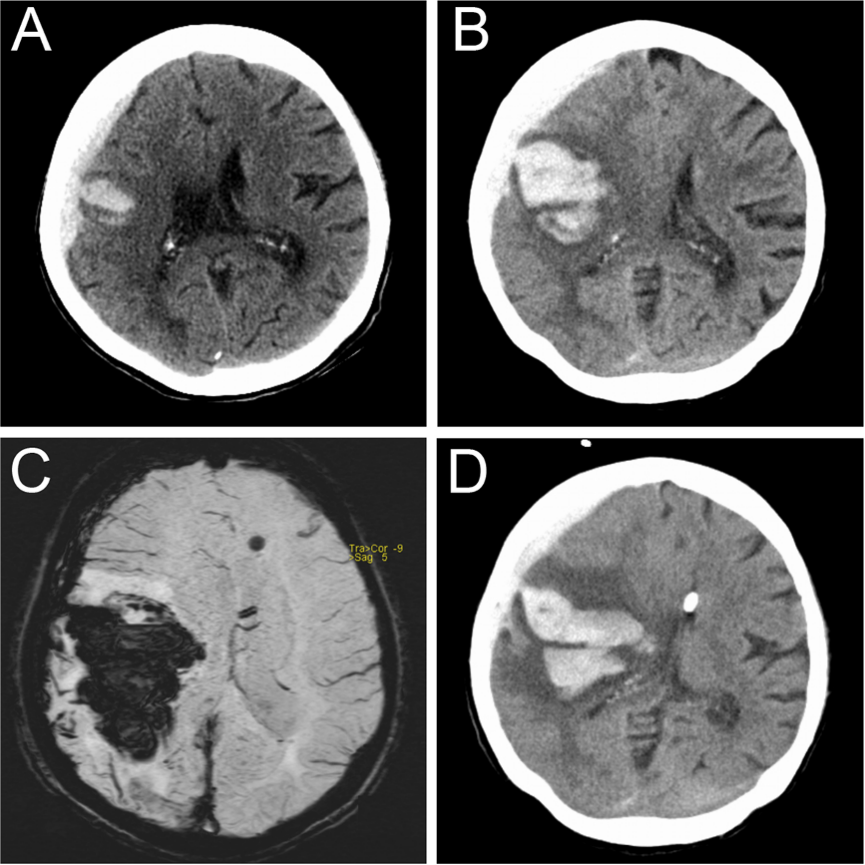
**Neuroradiological imaging results in the presented patient.** ***A***: CT-imaging on admission. ***B***: CT-control-imaging 4 hours later. ***C***: Susceptibility weighted MRI after 1 day. ***D***: CT-imaging 4 days after admission and before termination of osmotic therapy and therapeutic hypothermia.

A 77-year old woman was referred to our hospital because of acute hemiparesis of the left side. A cerebral CT scan revealed an intracerebral hemorrhage with a volume of 32 milliliter located in the right parietal cortex with concomitant right hemispheric subarachnoid blood accumulation (Figure 1A). Due to rapid worsening of the paresis and her consciousness, she was orotracheally intubated and started on imaging, Figure 1B) showed significant worsening. An external ventricular drainage was surgically put in place to prevent hydrocephalus and to control intracerebral pressure. Follow-up MRI revealed an increased size of the bleeding to 112 milliliter, large perifocal edema and a midline shift of 9 millimeter. Susceptibility weighted images revealed multiple microbleeds as a correlate of amyloid angiopathy as most probable cause of the hemorrhage (Figure 1C). There was no evidence for other causes of the initial event and for the expansion of the hematoma: Blood coagulation parameters were within normal limits, there was no history of arterial hypertension. Under the influcence of analgosedatives, blood pressure had to be supported by continuous infusion of norepinephrine and did not exceed a systolic pressure of 160 mmHg and a mean arterial blood pressure of 100 mmHg diastolic. Echocardiography was unremarkable.

**Figure 2:**
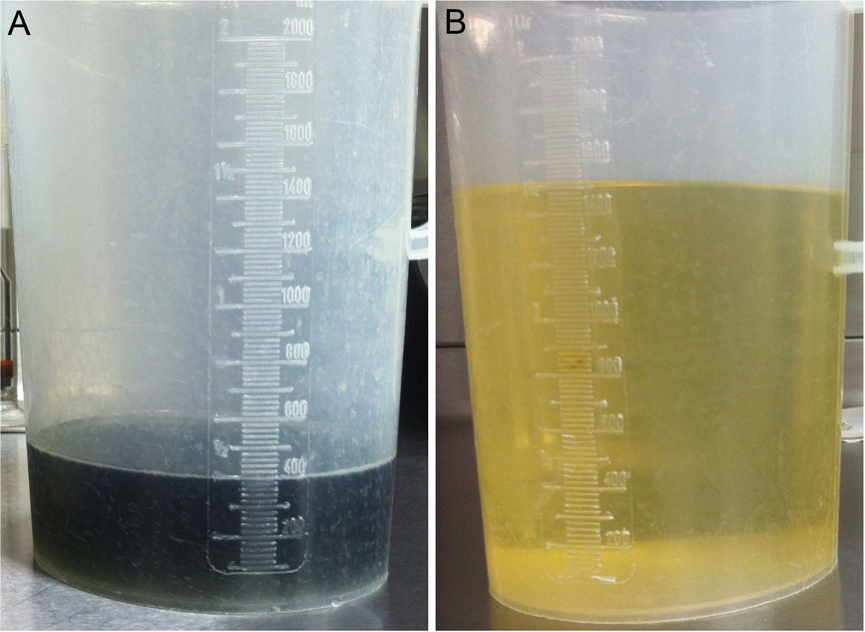
**Urine samples of the patient.** ***A***: Dark green discoloration 48 hours after the onset of propofol. ***B***: Color changed back to normal few hours after the end of propofol administration.

Due to the age of the patient, a decision was made against surgical hematoma evacuation and for aggressive medical treatment. To reduce edema, osmotic therapy with mannitol and hypertonic saline was started. Serum osmolality was thereby increased to 330 mosmol/kg with a sodium level of 150 mmol/l. As an additional anti-edema therapy, mild therapeutic hypothermia (35,0°C) was induced with an endovascular cooling catheter (Staykov et al. 2013).86 hours after the onset of analgosedatives and 16 hours after induction of hypothermia, we noted a green-brown discoloration of the patient’s urine (Figure 2A). Standard urine analysis was unremarkable (pH 6,5, protein neg., nitrite neg., wbc neg., ketone neg., bilirubine neg., blood neg.) and urine cultures were negative. Liver function parameters including bilirubin were normal. At the same time, obstipation in presence of enteral feeding via a nasogastral tube was treated with laxatives. Analgosedatives were switched to midazolame and sufentanil, and propofol (total amount of 14,000 mg, average infusion rate of 3.2 mg/kg/h) was discontinued. Within few hours, urine color changed back to normal (Figure 2B).Despite maximal therapy, CT follow-up after 3 days showed significant worsening with increased edema, mesencephalic compression and subfalcine and uncal herniation (Figure 1D). Due to poor prognosis, osmotic therapy was stopped and the patient died two days later due to cerebral herniation.

## Discussion

Urine discoloration due to propofol infusion has been described few times in the literature (Bodenham et al. 1987; Tan et al. 2008; Ku et al. 2011; Shioya et al. 2011; Barbara and Whalen 2012). Potential other causes include amitriptyline, indomethacin, cimetidine, metoclopramide, methocarbamol and promethazine as well as urinary tract infection or food coloring (Carpenito and Kurtz 2002; Ehrig et al. 1999; Pak 2004; Gillett and Burnett 2006; Tonseth et al. 2007; Bernante et al. 2003; Ananthanarayan and Fisher 1995). Few of these constellations reflect a harmful situation *per se* and neither health care staff nor relatives should be alarmed. If urine analysis is normal, the accountable drug should be identified by pausing medications that are known for this side effect.

To the best of our knowledge, this is the first report describing urine discoloration in a patient treated with osmotic therapy and mild hypothermia. Hypothermia is also known to increase the risk of tubular dysfunction, electrolyte loss and electrolyte disorders and this may be potentiated by the renal side effects of osmotic therapy (Brain Trauma Foundation 2007; Polderman et al. 2001a; Polderman et al. 2001b; Kaufman et al. 1993; Weinberg 1993). However, in our patient, creatinine and urea levels remained at baseline levels throughout her disease course and serial arterial blood analyses showed normal pH levels. Hypothermia reduces the metabolic rate by 7–9% per degree Celsius reduction of body temperature (Polderman 2004). Propofol is metabolized by hepatic and renal glucuronidation (Oda et al. 2001; McGurk et al. 1998). The mechanism of propofol-induced urine discoloration is supposed to be mediated by extrahepatic propofol glucuronidation due to decreased hepatic glucuronidation as a consequence of reduced liver enzyme function or diminished peristalsis (Shioya et al. 2011). The green color is a consequence of the excretion of the quinol derivates 4-(2,6-diisopropyl-1,4-quinol)-sulphate, 1- and 4-(2,6-diisopropyl-1,4)-glucuronide resulting from renal sulfo- and glucuroconjugation of propofol (Simons et al. 1988).

We assume that decreased liver enzyme function due to hypothermia, decreased enterohepatic circulation due to obstipation and a high-normal rate of propofol analgosedation may have provoked urine discoloration in our patient. Although urine discoloration is regarded as a benign side effect of propofol, we conclude that care should be taken in patients treated with hypothermia to maintain renal function and peristalsis. Future studies are needed to show interactions of hypothermia with the observed side effect of propofol.

### Consent

Due to the fatal outcome, written informed consent for the publication of this report could not be obtained from the patient; therefore all images are anonymized.
